# Olfactory Dysfunction and Its Relationship With Clinical Features of Parkinson's Disease

**DOI:** 10.3389/fneur.2020.526615

**Published:** 2020-10-16

**Authors:** Yangjie Zhou, Runcheng He, Yuwen Zhao, Yan He, Yacen Hu, Qiying Sun, Qian Xu, Jieqiong Tan, Xinxiang Yan, Beisha Tang, Jifeng Guo

**Affiliations:** ^1^Department of Neurology, Xiangya Hospital, Central South University, Changsha, China; ^2^Department of Geriatric Disorders, Xiangya Hospital, Central South University, Changsha, China; ^3^Laboratory of Medical Genetics, Central South University, Changsha, China; ^4^Key Laboratory of Hunan Province in Neurodegenerative Disorders, Central South University, Changsha, China; ^5^National Clinical Research Center for Geriatric Disorders, Changsha, China

**Keywords:** Parkinson's disease, olfactory dysfunction, hyposomia rating scale, Sniffin' Sticks test, non-motor symptom

## Abstract

**Objective:** To conduct an investigation into the reliability of assessing the olfactory function of patients with Parkinson's disease (PD) in a clinical setting of crowding patients in populated countries, such as China, by the hyposmia rating scale (HRS) and compare other non-motor features between patients with PD with olfactory dysfunction (PD-OD) and patients with PD without olfactory dysfunction (PD-NOD), according to the result of olfactory function assessed by the Sniffin' Sticks test.

**Methods:** A total of 320 patients with clinically confirmed or clinically possible PD were recruited. Olfactory function of all participants was assessed with the HRS and the Sniffin' Sticks test. Demographic data and clinical information were collected, and patients were evaluated using standardized assessment protocols. With reference to the Sniffin' Sticks test, the specificity, sensitivity, coincidence rate, and kappa value of the HRS was computed, and then its reliability was evaluated. We divided patients into PD-OD and PD-NOD groups based on the results of olfactory function assessed by the Sniffin' Sticks test. Clinical manifestations were compared between PD-OD and PD-NOD.

**Results:** The percentage of patients with OD determined by the Sniffin' Sticks test was 65.6%, and the percentage of those with OD was 55.6% when using the HRS measured olfactory function. With reference to the Sniffin' Sticks test, the specificity, sensitivity, coincidence rate, and kappa value of the HRS were 82.73, 75.71, 78.13%, and 0.55, respectively. The area under the receiver operating characteristic curve for the HRS was 0.793. There were no differences in demographic characteristics between the PD-OD and PD-NOD groups. The patients with hyposmia had more severe non-motor symptoms.

**Conclusion:** The HRS is of great value as a self-assessment scale for evaluating olfactory function, especially in PD patients over 55 years old. Moreover, PD patients with hyposmia have more severe non-motor features than PD patients without hyposmia, mainly in terms of mood and constipation.

## Introduction

Parkinson's disease (PD) is the second most common neurodegenerative disease. Environmental exposure and genetics are considered potent risk factors for PD (1, 2). A range of pathogenic mechanisms is likely to contribute to the progression of PD, such as protein aggregation, immune inflammation, oxidative stress, and mitochondrial dysfunction (3). The main clinical manifestations include motor symptoms, such as rest tremor, bradykinesia, rigidity, postural instability, and gait difficulties (4). In addition, there are some non-motor symptoms, including constipation, cognitive impairment, insomnia, hyposmia, psychiatric symptoms, autonomic dysfunction, pain, and so on (5). Among them, with a prevalence of 50–90%, olfactory dysfunction is one of the best characterized and most common non-motor features among PD patients (6). Moreover, some studies find that, before the onset of motor symptoms, there were olfactory impairments for several years, and people with olfactory dysfunction have an increased risk of developing PD (7–9). In addition, the study shows that the sensitivity and specificity of PD diagnosis could be improved if the assessment of motor symptoms and olfactory detection are combined (10). Hyposmia is used as an important support standard for diagnosing PD in the Movement Disorder Society (MDS) clinical diagnostic criteria for PD (11), and it might be a potential marker for predicting PD (12–15). Therefore, detecting olfactory function is important.

Olfactory tests can be divided into electrophysiological tests (e.g., odor event-related potentials), psychophysical tests (e.g., detection, identification, discrimination, and memory of odors), and psychophysiological tests (e.g., odorant-related respiratory changes) (16–18). Psychophysical tests, which are the most practical and the most widely used, mostly use some form of odor identification (for example, the Sniffin' Sticks test). Because it takes a long time to conduct the Sniffin' Sticks test to assess olfactory function and the procedure is relatively complicated, it is not used widely in the clinic. In China and other countries where a clinical setting of crowding patients is commonly seen, dozens of patients with PD can be seen every week. It is not practical to use the Sniffin' Sticks test to evaluate olfactory function. In 2012, Millar Vernetti et al. proposed a method for assessing olfactory function in patients with PD, which is simple, economical, time-saving, and reliable, called the hyposmia rating scale (HRS) (19).

This study aimed to conduct an investigation into the reliability of assessing the olfactory function of patients with PD in China by the HRS. In addition, we compare other non-motor features between PD patients with olfactory dysfunction (PD-OD) and those without olfactory dysfunction (PD-NOD) based on the olfactory function results evaluated by the Sniffin' Sticks test.

## Participants and Methods

### Parkinson's Disease Patients

Inclusion criteria: patients diagnosed with clinically confirmed or clinically possible PD according to MDS clinical diagnostic criteria for PD were recruited (11).

Exclusion criteria were chronic rhinitis, sinusitis, and chronic obstructive pulmonary disease; acute respiratory infection within the previous 3 weeks; prolonged or extensive exposure to volatile substances, such as cleaning supplies or sawdust, acid fumes, pesticides, herbicides, metal dust, and industrial solvents; severe head trauma or nasal surgery; drug abuse; and neuropsychiatric diseases, such as dementia or schizophrenia.

According to the calculation formula of sample size for diagnostic research, we got a minimum sample size of 81. From February 2017 to March 2018, a total of 320 patients with PD were consecutively enrolled in the Department of Neurology, Xiangya Hospital, Central South University. All patients signed informed consent forms, and they were all registered in the Parkinson's Disease and Movement Disorders Multicenter Database and Collaborative Network in China (PD-MDCNC) database. This study was approved by the ethics committee of Xiangya Hospital of Central South University.

### Collection of Demographic Information

Demographic variables for all PD participants include gender, age, body mass index (BMI), age at onset, disease duration, and education.

### Evaluation of Olfactory Function by the Sniffin' Sticks Test

The Sniffin' Sticks test, which was developed, designed, and produced by Burghart Messtechnik GmbH, Germany (website: www.burghart-mt.de), was used to evaluate olfactory function for all participants (20). There are a total of 112 sticks, of which 48 sticks are used for testing the olfactory threshold (T), 48 for olfactory discrimination (D), and 16 for olfactory identification (I).

Determination of T scores: Before the test, the patients smell the odor associated with stick No. 1 (containing the highest concentration of n-butanol), and then the subjects close their eyes for the test. Starting from the lowest concentration (No. 16), the concentration gradient is gradually increased until the subjects correctly perceive the concentration. When the subjects correctly perceive the red stick (containing n-butanol) twice in succession, it is recorded as the accurate concentration level. The test is then performed with a lower first-order concentration until the patients are not aware of the odor. Then, this concentration is the reversal point of the next series of tests, and the concentration level is recorded. The concentration is then stepped up to the first-order concentration until the subjects are able to correctly perceive the red stick, and the reversal point level is recorded again. This reciprocating cycle is performed such that, when seven reversal points are found, the olfactory test ends, and the average of at least four reversal point concentration levels is taken as the value for the T score.

Determination of D scores: The patients are asked to discriminate the target odor, which is different from the other two odors. The number of different odors that the subjects correctly discriminate is recorded as the D score.

Determination of I scores: The patients are asked to choose one of the four given answers that match the best description of the odor they smell. The number of correctly identified odors for each subject is recorded as the I score.

The TDI score is used to assess overall olfactory function and is calculated as the sum of the T, D, and I scores.

Olfactory dysfunction is defined on the basis of a study of olfactory function in more than 3,000 subjects with the Sniffin' Sticks test (21). Patients aged from 16 to 35 with TDI scores ≤ 30.3 points are diagnosed with OD, and patients aged from 36 to 55 with TDI scores ≤ 27.3 points are diagnosed with OD. OD is diagnosed in patients aged older than 55 with TDI scores ≤ 19.6 points. According to this, we divided patients into two groups. The age groups were divided into 36–55 and more than 55.

### Assessing Olfactory Function With the HRS

In 2012, Millar Vernetti et al. developed a scale named HRS for assessing olfactory function in PD patients. The HRS consists of 6 items, each with a score ranging from 0 to 4 for a total score of 24 (19). According to Patricio Millar Vernetti, the optimal cutoff value on the HRS is 22.5. Therefore, patients with total HRS scores ≤ 22 and ≥23 are defined as having OD and no OD, respectively.

The specificity, sensitivity, positive predictive value, negative predictive value, positive likehood ratio, negative likehood ratio, coincidence rate, and kappa value for the HRS are calculated with reference to the results of the Sniffin' Sticks test.

### Assessments of Clinical Symptoms of PD

The non-motor symptoms scale (NMSS) is used to evaluate non-motor symptoms, including cardiovascular, mood, gastrointestinal symptoms, urinary symptoms, and so on. For the evaluation of cognitive impairment, the participants are assessed using the Mini-Mental State Examination (MMSE). The Epworth sleepiness scale (ESS) is applied to evaluate excessive daytime sleepiness. The rapid eye movement (REM) sleep behavior disorder questionnaire–Hong Kong (RBDQ-HK) and Hamilton rating scale for depression (HAMD-17) were applied to assess REM sleep behavior disorder (RBD) and depression, respectively. The presence of constipation is assessed by the Rome III functional constipation diagnostic criteria (22).

### Statistical Analysis

SPSS version 19.0 was used to analyze the data with *P* ≤ 0.05 indicating significance.

Means and standard deviations are used to describe continuous variables while percentages and frequencies are used to describe categorical variables. The *t*-test or chi-square test is used to compare the demographics; scores of olfactory functions, including T, D, I, and TDI scores; and clinical characteristics between PD patients with and without olfactory dysfunction. Logistic regression analysis is used to evaluate the factors associated with olfactory dysfunction in the PD patients. In the univariate logistic regression model, after adjusting for age, sex, and duration of disease, variables with *P* < 0.1 are considered in the multivariate logistic model. A likelihood ratio (LR) stepwise procedure is used in the multivariate logistic model.

## Results

### Comparisons of the Sniffin' Sticks Test and HRS in Terms of Assessing OD in Patients With PD

**Figure 1:**
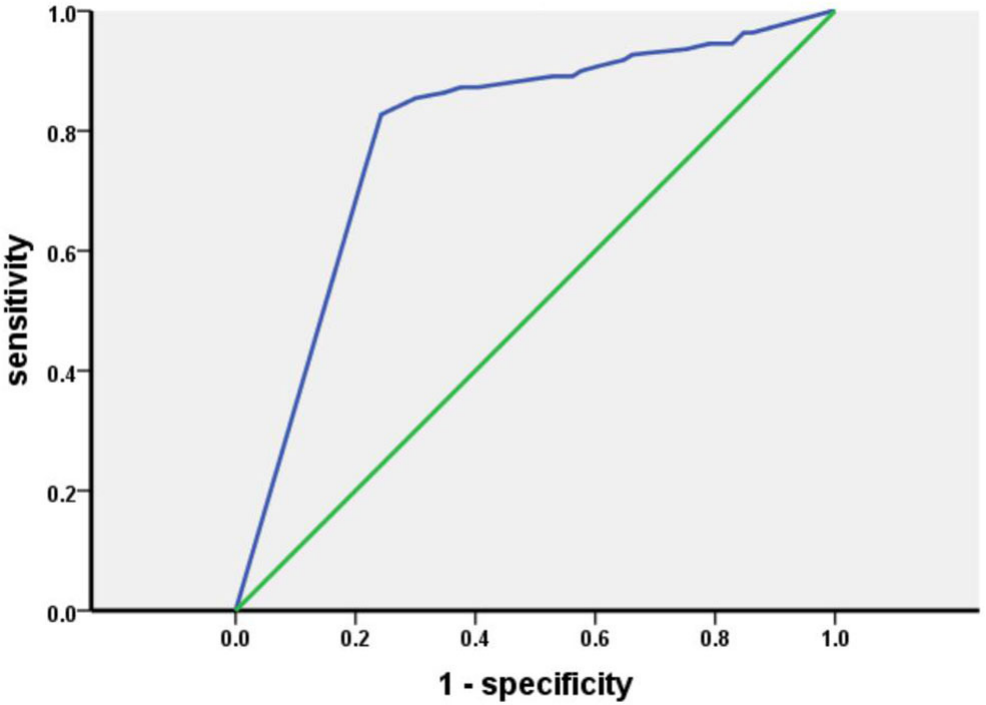
ROC curve for the HRS.

The results of the olfactory function assessments evaluated by the Sniffin' Sticks test and HRS in patients with PD are shown in Table 1. In this study, the percentage of patients with OD evaluated by the Sniffin' Sticks test was 65.6%, and the percentage of patients with OD was 55.6% when using the HRS-measured olfactory function. With reference to the Sniffin' Sticks test, the specificity, sensitivity, positive predictive value, negative predictive value, positive likehood ratio, and negative likehood ratio of the HRS are 82.73, 75.71, 89.32, 64.08%, 4.38, and 0.29, respectively. The coincidence rate of HRS is 78.13%, and the kappa value is 0.55. The area under the receiver operating characteristic (ROC) curve (AUC) for the HRS is 0.793 (*P* < 0.05) (Figure 1).

**Table 1:** Results of olfactory function evaluated by the Sniffin' Sticks test and HRS.

**HRS**	**Sniffin' Sticks test**		**Total**
	**OD**	**NOD**	
OD	159	19	178
NOD	51	91	142
Total	210	110	320

*HRS, hyposmia rating scale; OD, olfactory dysfunction; NOD, non-olfactory dysfunction*.

In PD patients older than 55, the results of the olfactory function assessments measured by the Sniffin' Sticks test and HRS are shown in Supplementary Table 1. The percentages of patients with OD based on the Sniffin' Sticks test and HRS are 57.2 and 57.7%, respectively. With reference to the Sniffin' Sticks test results, the specificity, sensitivity, and coincidence rate of the HRS are 80.72, 86.49, 84.02%, respectively. The AUC for the HRS is 0.85 (*P* < 0.05) (Supplementary Figure 1). We recalculated the HRS cutoff value and found it was still 22.5 points.

In PD patients aged from 36 to 55, the results of olfactory function assessments evaluated by the Sniffin' Sticks test and HRS are shown in Supplementary Table 2. The percentages of patients with OD based on the Sniffin' Sticks test and HRS are 78.6 and 52.4%, respectively. With reference to the Sniffin' Sticks test results, the specificity, sensitivity, and coincidence rate of the HRS are 88.89, 63.63, 69.05%, respectively. The AUC for the HRS is 0.747 (*P* < 0.05) (Supplementary Figure 2). We recalculated the HRS cutoff value and found it was still 22.5 points.

### Evaluation of Olfactory Function Between PD-OD and PD-NOD

For the 320 PD patients, the average TDI, T, D, and I scores are 19.3 (7.7), 5.0 (3.5), 7.4 (3.2), and 6.9 (3.0) points, respectively. Further comparisons reveal that the means of the TDI, T, D, and I scores in the PD-OD group are all significantly lower than those in the PD-NOD group (*P* < 0.01) (Table 2).

**Table 2:** Demographic variables and clinical symptoms for PD patients, PD-OD, and PD-NOD.

**Variables**	**PD (*N* = 320)**	**PD-OD (*N* = 210)**	**PD-NOD (*N* = 110)**	***P***
Age [mean ± SD]^a^	59.43 ± 9.54	58.89 ± 10.05	60.49 ± 8.47	0.133
Age at onset [mean ± SD]^a^	53.93 ± 10.16	53.24 ± 10.81	55.23 ± 8.75	0.077
Duration of disease [mean ± SD]^a^	5.57 ± 3.96	5.73 ± 4.18	5.26 ± 3.50	0.320
Male [patients (%)]^b^	165 (51.6)	112 (53.3)	53 (48.2)	0.411
BMI [mean ± SD]^a^	22.99 ± 3.05	22.80 ± 2.91	23.35 ± 3.30	0.125
Education [patients (%)]^b^				0.896
Primary school	222 (69.4)	143 (68.1)	79 (71.8)	
Middle school and above	91 (28.4)	60 (28.6)	31 (28.2)	
TDI [mean ± SD]^a^	19.3 ± 7.7	16.0 ± 7.0	25.7 ± 4.3	**<0.001**
T [mean ± SD]^a^	5.0 ± 3.5	3.8 ± 2.9	7.5 ± 3.2	**<0.001**
D [mean ± SD]^a^	7.4 ± 3.2	6.4 ± 3.2	9.2 ± 2.5	**<0.001**
I [mean ± SD]^a^	6.9 ± 3.0	5.8 ± 2.8	9.0 ± 2.2	**<0.001**
NMSS total [mean ± SD]^a^	36.69 ± 26.14	39.60 ± 28.28	31.15 ± 20.62	**0.003**
NMSS domain 1 [mean ± SD]^a^	0.73 ± 1.62	0.78 ± 1.75	0.64 ± 1.34	0.450
NMSS domain 2 [mean ± SD]^a^	8.62 ± 7.39	8.78 ± 7.54	8.31 ± 7.16	0.590
NMSS domain 3 [mean ± SD]^a^	6.44 ± 8.38	7.14 ± 9.17	5.12 ± 6.57	**0.024**
NMSS domain 4 [mean ± SD]^a^	1.04 ± 2.39	0.98 ± 2.40	1.15 ± 2.40	0.539
NMSS domain 5 [mean ± SD]^a^	3.76 ± 4.02	4.02 ± 4.16	3.27 ± 3.71	0.115
NMSS domain 6 [mean ± SD]^a^	3.93 ± 4.70	4.13 ± 4.79	3.55 ± 4.53	0.300
NMSS domain 7 [mean ± SD]^a^	5.80 ± 5.92	6.15 ± 6.20	5.15 ± 5.33	0.151
NMSS domain 8 [mean ± SD]^a^	0.67 ± 2.56	0.74 ± 2.70	0.55 ± 2.33	0.552
NMSS domain 9 [mean ± SD]^a^	5.76 ± 5.62	7.0 ± 6.01	3.41 ± 3.87	**<0.001**
MMSE score [mean ± SD]^a^	27.08 ± 3.02	26.91 ± 3.14	27.39 ± 2.77	0.181
ESS score [mean ± SD]^a^	7.93 ± 5.88	8.19 ± 6.16	7.43 ± 5.34	0.257
EDS [patients (%)]^b^	122 (38.1)	84 (40.0)	38 (34.5)	0.334
HAMD-17 score [mean ± SD]^a^	5.77 ± 5.21	6.21 ± 5.68	4.96 ± 4.54	**0.036**
Depression [patients (%)]^b^	87 (27.2)	61 (29.0)	26 (23.6)	0.292
RBD [patients (%)]^b^	141 (44.1)	97 (46.2)	44 (40.0)	0.286
Constipation [patients (%)]^b^	136 (42.5)	108 (51.4)	28 (25.5)	**<0.001**

a *t-test*.

b *Chi-squared test*.

*TDI, Threshold + Discrimination + Identification; T, Threshold; D, Discrimination; I, Identification*.

*SD, standard deviation; NMSS, Non-motor symptoms scale; MMSE, Mini-Mental state examination; ESS, Epworth sleepiness scale; EDS, Excessive daytime sleepiness; HAMD-17 score, Hamilton rating scale for depression; RBD, Rapid eye movement sleep behavior disorder; NMSS domain 1: cardiovascular system; NMSS domain 2, sleep and fatigue; NMSS domain 3, emotion and cognition, NMSS domain 4, perceptions and hallucinations; NMSS domain 5, attention and memory; NMSS domain 6, astrointestinal system; NMSS domain 7, urinary system; NMSS domain 8, sexual function; NMSS domain 9, pain, olfactory function, sweating and weight change. The bold values represent P < 0.05 which means there are statistical significance*.

### Demographic Information for All PD Patients and the PD-OD and PD-NOD Groups

Demographic variables are compared between the PD-OD and PD-NOD groups and include gender, BMI, age, age at onset, disease duration, and education (Table 2). The percentage of males in this study is 51.6%. The mean age of all PD patients is 59.49 ± 9.52 years. The mean age at onset and duration of disease are 53.93 ± 10.16 and 5.57 ± 3.96 years, respectively. For educational level, the percentage of patients with primary school education is 69.4%, and the percentage for those with a middle school level of education and above is 28.4%. The mean BMI is 22.99 ± 3.05 kg/m^2^. There are no significant differences between the PD-OD and PD-NOD groups in demographic variables (*P* > 0.05).

In patients older than 55, the average age of the PD-OD patients is higher than that of the PD-NOD patients (*P* < 0.05), and patients aged from 36 to 55, show no difference in age across groups. For other demographic variables, there are no significant differences between the two groups regardless of age stratification (Table 3).

**Table 3:** Demographic variables for PD-OD and PD-NOD groups stratified by age.

**Variables**	**Age** **>** **55**		**Age from 36 to 55**		**P1**	**P2**
	**PD-OD (*N* = 111)**	**PD-NOD (*N* = 83)**	**PD-OD (*N* = 99)**	**PD-NOD (*N* = 27)**		
Age [mean ± SD]^a^	67.11 ± 5.27	64.33 ± 5.35	49.67 ± 4.68	48.70 ± 4.43	**<0.0010**	0.340
Age at onset [mean ± SD]^a^	61.04 ± 7.58	58.81 ± 6.31	44.51 ± 6.23	44.22 ± 5.32	**0.031**	0.830
Duration of disease [mean ± SD]^a^	6.07 ± 4.49	5.52 ± 3.48	5.34 ± 3.80	4.48 ± 3.51	0.351	0.291
Male [patients (%)]^b^	57 (51.4)	37 (44.6)	55 (55.6)	16 (59.3)	0.385	0.828
BMI [mean ± SD]^a^	22.69 ± 3.06	23.21 ± 3.28	22.92 ± 2.75	23.78 ± 3.39	0.254	0.176
Education [patients (%)]^b^					1.000	0.823
Primary school	83 (74.8)	63 (75.9)	60 (60.6)	16 (59.3)		
Middle school and above	25 (22.5)	20 (24.1)	35 (35.4)	11 (40.7)		

a *t-test*.

b *Chi-squared test*.

*P1, P-value of comparison between PD-OD and PD-NOD older than 55*.

*P2, P-value of comparison between PD-OD and PD-NOD aging from 36 to 55. The bold values represent P < 0.05 which means there are statistical significance*.

### Comparisons of Clinical Features Between the PD-OD and PD-NOD Groups

The non-motor symptom information for all PD patients as well as the data stratified by PD-OD and PD-NOD are shown in Table 2. Comparing non-motor symptoms between the PD-OD and PD-NOD groups, we find that there are no differences between PD-OD and PD-NOD in MMSE scores and ESS scores (*P* > 0.05). In comparison with the PD-NOD group, the PD-OD group does not show a significantly different proportion of patients with RBD and EDS (*P* > 0.05). Although the percentage of patients with depression is not different between the PD-OD and PD-NOD groups (*p* > 0.05), the HAMD-17 scores are greater in the patients with PD-OD than in those with PD-NOD (*p* = 0.046), which is consistent with the results on the NMSS domain 3 describing mood (*p* = 0.039). In terms of the NMSS, the NMSS total scores and NMSS domain 9 scores, including olfactory function, are higher in the PD-OD groups than in the PD-NOD group (*P* < 0.05). However, there are no differences in NMSS domains 1, 2, and 4 to 8 (*p* > 0.05). Regarding constipation, the proportion of patients with constipation is significantly greater in the PD-OD group than in the PD-NOD group (*P* < 0.05).

### Logistic Regression Analysis of Factors Associated With Olfactory Dysfunction

**Figure 2:**
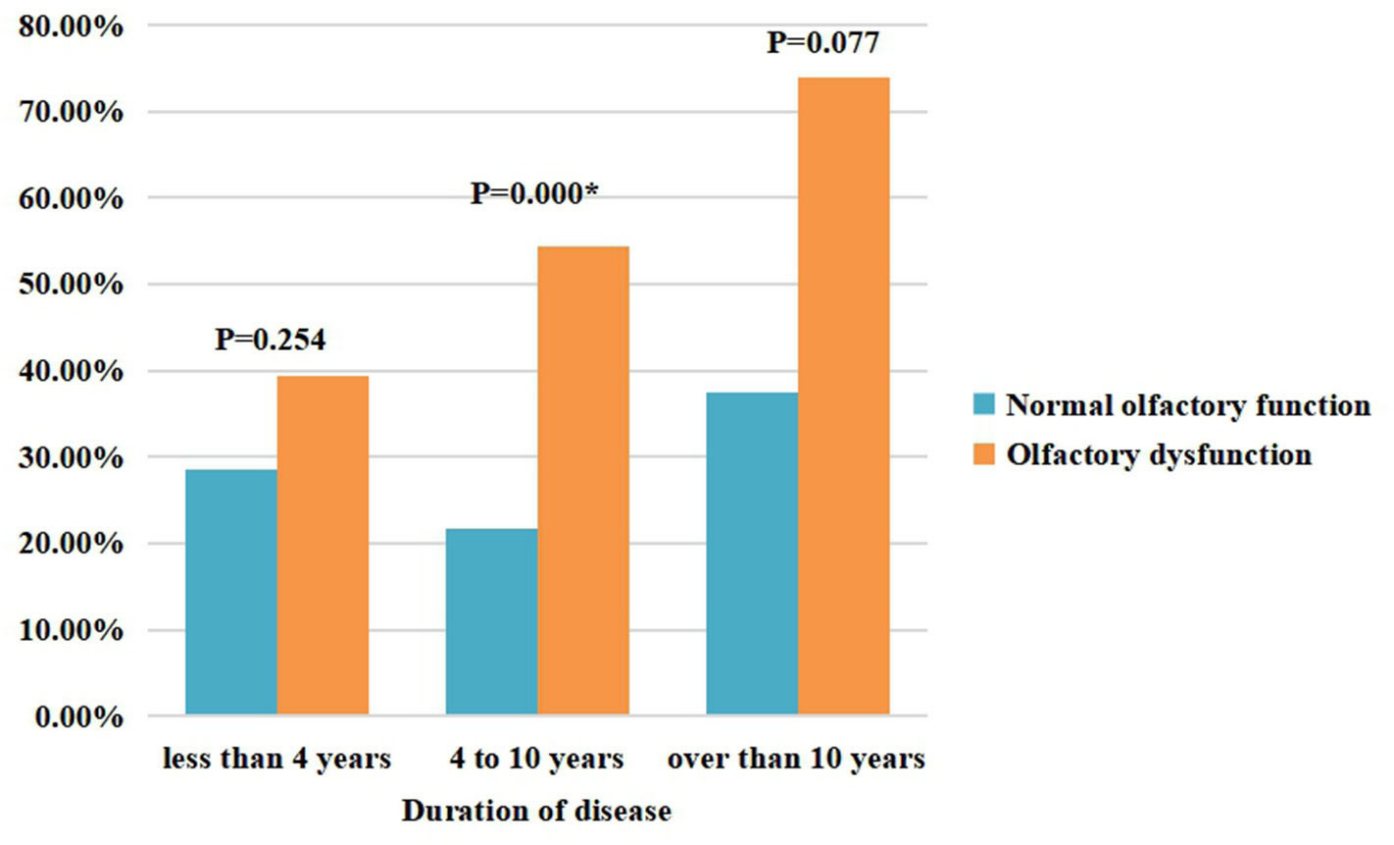
Constipation in the PD-OD and PD-NOD groups.

Table 4 shows the factors associated with olfactory dysfunction in patients with PD. The results indicate that constipation is associated with olfactory dysfunction in patients with PD. Figure 2 further compares constipation between the PD-OD and PD-NOD groups while stratifying the PD patients according to disease duration.

**Table 4:** Logistic regression analysis of factors associated with olfactory dysfunction.

**Variables**	Univariate *P*-value^*^	**Multivariate OR (95% CI)**	**Multivariate *P*-value**
Age	0.149^†^	–	Not included
Age at onset	0.169^†^	–	Not included
Duration of disease	0.308^§^	–	Not included
Male	0.423^‡^	–	Not included
BMI	0.126	–	Not included
Education	0.931	–	Not included
NMSS domain 1	0.238	–	Not included
NMSS domain 2	0.517	–	Not included
NMSS domain 3	**0.039**	–	NS
NMSS domain 4	0.534	–	Not included
NMSS domain 5	0.056	–	NS
NMSS domain 6	0.199	–	Not included
NMSS domain 7	0.075	–	NS
NMSS domain 8	0.570	–	Not included
MMSE score	0.062	–	NS
ESS score	0.209	–	Not included
EDS	0.253	–	Not included
HAMD-17 score	0.066	–	NS
Depression	0.361	–	Not included
RBD	0.227	–	Not included
Constipation	**<0.001**	3.252 (1.949–5.426)	**<0.001**

* *Adjusted for age, gender and duration of disease (except where otherwise noted)*.

† *Adjusted for gender and duration of disease*.

§ *Adjusted for age and gender*.

‡ *Adjusted for age and duration of disease. The bold values represent P < 0.05 which means there are statistical significance*.

## Discussion

It is important to detect olfactory dysfunction, which is one of the best characterized and most common non-motor features among PD patients.

In our study, hyposmia is assessed by two means. Using the Sniffin' Sticks test to evaluate olfactory function, 210 patients (65.6%) are found to have olfactory dysfunction, which is consistent with a previous report (6, 23, 24). This indicates that hyposmia is one of the most common non-motor symptoms in patients with PD. The number of patients with hyposmia detected by the HRS is 178 (55.6%), which is lower than that found by the Sniffin' Sticks test, indicating that the patients had not realized that their olfactory function was failing. Interestingly, we find that TDI scores in patients with hyposmia diagnosed by the two means are significantly lower than those in patients who are diagnosed with hyposmia and normal olfactory function by the Sniffin' Sticks test and HRS, respectively (Supplementary Table 3). This signifies that patients did not realize that their olfactory function had decreased in the early stage of olfactory dysfunction. With reference to the Sniffin' Sticks test, the specificity, sensitivity, positive predictive value, negative predictive value, positive likehood ratio, and negative likehood ratio of the HRS are 82.73, 75.71, 89.32, 64.08%, 4.38, and 0.29, respectively. The coincidence rate of HRS is 78.13%, and the kappa value is 0.55. These results reveal that the use of the HRS to assess olfactory function in our population is highly consistent with the evaluation of olfactory function by the Sniffin' Sticks test. The AUC of HRS is 0.793, showing that HRS has a medium diagnostic value. Because the cutoff values of the Sniffin' Sticks test are different across ages, we further evaluate the use of the HRS after stratifying by age. Considering that olfactory function is associated with aging, and olfactory function in the elderly population declines with increasing age, more granular segregation of age groups is better. In our study, the age groups are divided into 36–55 and more than 55 according to the study on the basis of which olfactory dysfunction is defined. In the patients older than 55, with reference to the Sniffin' Sticks test, the specificity, sensitivity, and coincidence rate of the HRS are 80.72, 86.49, 84.02%, respectively. In the patients aged 36–55, with reference to the Sniffin' Sticks test, the specificity, sensitivity, and coincidence rate of the HRS were 88.89, 63.63, and 69.05%, illustrating that the HRS is more suitable for olfactory function assessment in patients older than 55.

In this study, the TDI, T, D, and I scores in the PD-OD group are all significantly lower than those in the PD-NOD group (Table 2), suggesting that PD-OD is characterized by an overall decline in olfactory threshold, discrimination, and identification.

There is no significant difference in the demographic characteristics between the PD-OD and PD-NOD groups. Previous studies show that PD-OD patients are older than PD-NOD patients (25, 26). We analyzed these studies and find that they did not use different cutoff values based on age. With advancing age, the ability of humans to detect and discriminate odors declines (27). Selecting a uniform cutoff value may cause patients with olfactory dysfunction to have an older age. We then stratified PD patients according to age and separately analyzed them. We find that the PD-OD patients are older than the PD-NOD patients among the patients older than 55 years (*P* < 0.05). Although there is no significant difference in age between the PD-OD and PD-NOD groups in patients aged 36–55, the average age in the PD-OD group is slightly greater than that in the PD-NOD group. This indicates that the age of patients with hyposmia is indeed older than the age of patients without hyposmia. This is also consistent with the result of Murphy's study that olfactory function in the elderly population declines with increasing age (28).

Comparing the clinical features of the PD-OD and PD-NOD groups, we find that the PD-OD and PD-NOD groups are significantly different in terms of mood, which could be concluded from the domain 3 score of the NMSS and the Hamilton Depression Scale score. Many studies show that patients who lose their sense of smell are more likely to have depression, and there is a close relationship between olfactory dysfunction and depression, which may be related to the fact that the olfactory conduction pathway affects the serotonin system in the body and emotional centers, such as the hippocampus and amygdala (29–31). However, when we conduct multivariate logistic regression analysis, we do not find an association between depression and olfactory dysfunction. Perhaps we need more patients for further study. Studies show that olfactory dysfunction has a certain relationship with cognitive decline (32, 33), and olfactory dysfunction can even be regarded as an early marker of cognitive decline (34, 35). A study shows that patients with PD-MCI exhibit atrophy in the right entorhinal cortex compared to PD-NC (36). In our study, there is no significant statistical difference between the PD-OD and PD-NOD groups in MMSE scores, but the MMSE score in the PD-OD group tends to be lower than that in the PD-NOD group. Whether there are differences in cognitive function between PD patients with and without hyposmia may require further research by comparing across different cognitive domains. In addition, the proportion of constipation is significantly higher in the PD-OD group than in the PD-NOD group (*P* < 0.05). This is consistent with the finding that hyposmia and gastrointestinal symptoms are correlated (26). The Parkinson's Associated Risk Study (PARS) confirms that PD patients with olfactory dysfunction are more likely to endure non-motor symptoms, such as constipation (37). This may be related to the fact that early sites of Lewy pathology are the olfactory bulb and enteric plexus of the stomach (38, 39). In addition, non-motor symptoms in PD patients are associated with dysfunction of the microbiota-gut-brain axis. Gut microbiota mediates bidirectional communication between the central nervous system (CNS) and enteric nervous system (ENS) through integrated immunological, neuroendocrine, and neurological processes (3). It is shown that α-syn can travel from gut to brain or from brain to gut via diffusion (40, 41).

In summary, the HRS is of great value as a self-assessment scale for assessing olfactory function, especially in PD patients over 55. Olfactory dysfunction is common in patients with PD, which can account for 65.6% of this population, and is characterized by an overall decline in olfactory threshold, discrimination, and identification. In addition, the non-motor manifestations in PD patients with olfactory dysfunction are more severe than those without olfactory dysfunction, mainly in terms of mood and constipation.

## Data Availability Statement

The datasets generated for this study are available on request to the corresponding author.

## Ethics Statement

The studies involving human participants were reviewed and approved by Ethics Committee of Xiangya Hospital of Central South University. The patients/participants provided their written informed consent to participate in this study.

## Author Contributions

QS, JG, and BT conceived the study. YZho, RH, YZha, YHe, YHu, and QS carried out the analysis and interpretation of the data. JT, QX, XY, BT, and JG discussed and revised the manuscript. All authors gave the final approval for the manuscript to be published.

## Conflict of Interest

The authors declare that the research was conducted in the absence of any commercial or financial relationships that could be construed as a potential conflict of interest.
