# Supplementary material for: Olfactory Dysfunction and Its Relationship With Clinical Features of Parkinson's Disease
Source: Front Neurol. 2020 Oct 16;11:526615. doi: 10.3389/fneur.2020.526615 (PMC7596377; doi:10.3389/fneur.2020.526615)
Supplement: Supplementary file 1 [file Data_Sheet_1.docx]

sTable1 Results of olfactory function evaluated by Sniffin’ Sticks test and HRS in patients older than 55

| HRS | Sniffin’ Sticks test | | total |
| --- | --- | --- | --- |
|  | OD | NOD |  |
| OD | 96 | 16 | 112 |
| NOD | 15 | 67 | 82 |
| total | 111 | 83 | 194 |

HRS: hyposmia rating scale, OD: olfactory dysfunction, NOD: non-olfactory dysfunction

sTable2 Results of olfactory function evaluated by Sniffin’ Sticks test and HRS in patients aging from 36 to 55

| HRS | Sniffin’ Sticks test | | total |
| --- | --- | --- | --- |
|  | OD | NOD |  |
| OD | 63 | 3 | 66 |
| NOD | 36 | 24 | 60 |
| total | 99 | 27 | 126 |

HRS: hyposmia rating scale, OD: olfactory dysfunction, NOD: non-olfactory dysfunction

sTable3 Comparison of olfactory function and demographic variables between PD-OD (+/+) and PD-OD (+/-)

| Variables | PD-OD（+/+） | PD-OD（+/-） | P |
| --- | --- | --- | --- |
| TDI [mean±SD] | 14.43±6.96 | 20.92±4.34 | **<0.001** |
| T[mean±SD] | 3.19±2.89 | 5.55±2.19 | **<0.001** |
| D[mean±SD] | 5.89±3.18 | 8.06±2.59 | **<0.001** |
| I[mean±SD] | 5.35±2.79 | 7.31±2.28 | **<0.001** |
| Age[mean±SD] | 60.34±9.78 | 54.71±9.66 | **<0.001** |
| Age at onset[mean±SD] | 54.45±10.19 | 49.47±11.88 | **0.004** |
| Duration of disease[mean±SD] | 5.89±3.64 | 5.24±5.57 | 0.334 |
| Male [patients (%)] | 89（55.9） | 23（45.1） | 0.199 |

TDI:Threshold+Discrimination+Identification, T: Threshold, D: Discrimination, I: Identification,

SD: standard deviation, PD-OD (+/+):OD diagnosed by the Sniffin’ Sticks test and HRS,

PD-OD (+/-): OD diagnosed by the Sniffin’ Sticks test and NOD diagnosed by HRS


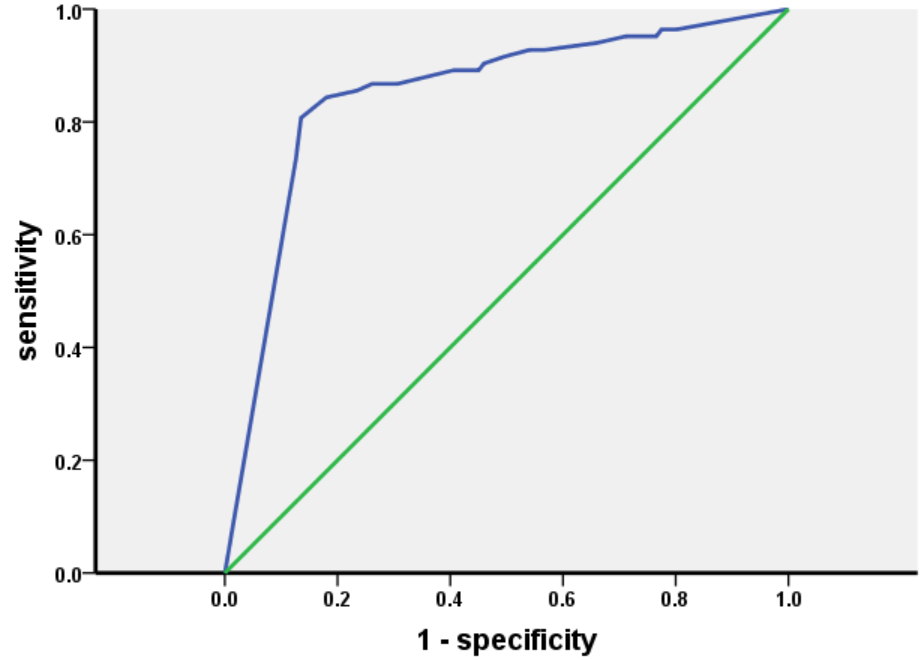


sFigure1 Receiver Operating Characteristic (ROC) curve for the HRS in patients older than 55


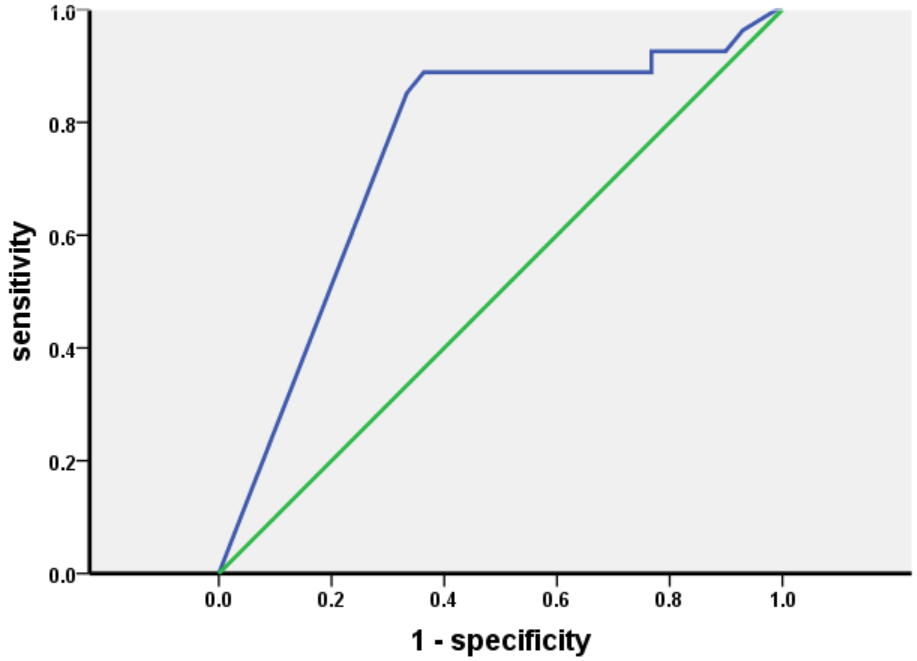


sFigure2 Receiver Operating Characteristic (ROC) curve for the HRS in patients aging from 36 to 55
